# A Rare Case of Lower Limb Sarcoma With BCOR-CCNB3 Mutation: Diagnosis and Treatment

**DOI:** 10.7759/cureus.35389

**Published:** 2023-02-23

**Authors:** Ansh Kedia, Gurpreet Singh, Subhangi Parmar, Hima Varsha, Tamara Tango, Pahel Agarwal, Sweta Sahu, Dharmesh R Chauhan

**Affiliations:** 1 Medicine, Institute of Medical Sciences, Banaras Hindu University, Varanasi, IND; 2 Medicine, Government Medical College & Hospital, Chandigarh, IND; 3 Internal Medicine, Gujarat Cancer Society (GCS) Medical College, Ahmedabad, IND; 4 Medical School, NRI Medical College & General Hospital, Chinakakani, IND; 5 Neurosurgery, Faculty of Medicine, Universitas Indonesia, Jakarta, IDN; 6 Internal Medicine, Bhaskar Medical College, Hyderabad, IND; 7 Surgery, Jagadguru Jayadeva Murugarajendra (JJM) Medical College, Davanagere, IND; 8 General Surgery, Government Medical College Surat, Surat, IND

**Keywords:** tumours, sarcoma, ewing-like sarcoma, lower limb sarcoma, bcor-ccnb3

## Abstract

In the past, BCOR-CCNB3 sarcoma was believed to be comparable to Ewing's sarcoma; however, current research has proven that it is, in reality, a distinct type of the illness, now classified as a distinct entity under undifferentiated round cell sarcomas. This tumour most frequently affects teenagers and young adults, and it is more prevalent in men. It can form in both bone and soft tissue, and it appears most frequently in the pelvis, lower extremities, and paraspinal region. Here, we describe a case of a soft tissue tumour in the proximal posterior portion of the right thigh of a 12-year-old male that was subsequently shown to be a BCOR-CCNB3 fusion using genetic analysis.

## Introduction

Undifferentiated small round cell sarcoma has been defined by the World Health Organization in 2020 as a fairly recently defined category of bone and soft tissue sarcoma [1]. This group, which was formerly referred to as Ewing-like sarcoma, includes small round cell sarcoma and Ewing sarcoma. The most common type of Ewing sarcoma is caused by the fusion of the FLI1 and EWS RNA-binding protein 1 (EWSR1) genes, which is caused by the chromosomal translocation anomaly t(11;22) (q24;q12) [2]. The so-called "Ewing-like sarcoma" is a pugnacious sarcoma that resembles Ewing sarcoma physically but lacks the typical fusion of EWSR1 and erythroblast transformation-specific (ETS) family genes, such as FLI1 [3]. It is characterised by small, spherical tumour cells that originate from bone and soft tissue. Ewing-like sarcoma, also referred to as undifferentiated small round cell sarcoma, has been recognised over the past two decades to typically express three distinct genetic characteristics: capicua transcriptional repressor (CIC)-rearranged sarcoma; BCL6 corepressor (BCOR)-rearranged sarcoma; and round cell sarcoma with EWSR1-non-ETS fusion [4]. Sixty to seventy per cent of undifferentiated small round cell sarcomas without EWSR1-ETS gene fusion are CIC-rearrangement sarcomas [5]. Molecular evidence suggests that these subtypes of sarcoma are biologically separate from Ewing sarcoma. BCOR-rearranged sarcomas have been documented predominantly in bone and infrequently in extraosseous tissues, affecting the kidney, neck, chest wall, and soft palate, with higher morbidity noted in male adolescents and children [6-8].

## Case presentation

**Figure 1:**
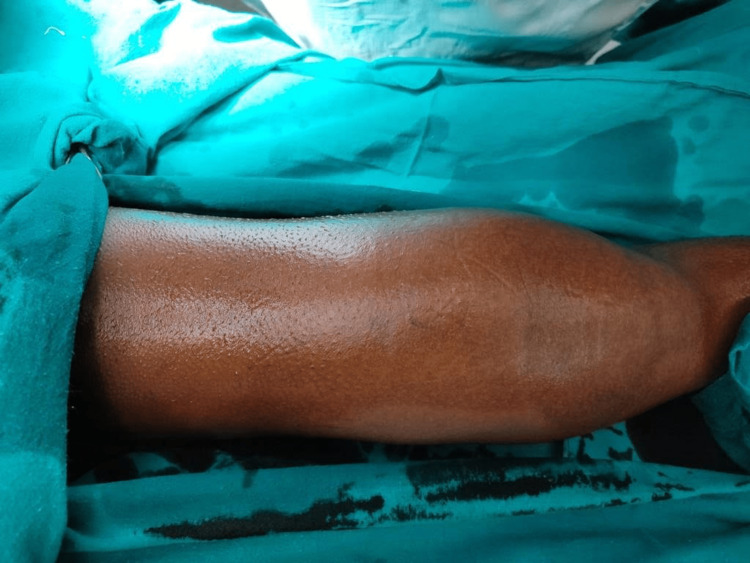
Non-tender, elastic, soft mass of size 30 centimetres x 15 centimetres

A 12-year-old boy presented with a one-year history of a gradually expanding mass in the posterior aspect of the right thigh. There were no reports of pain or difficulty walking. Physical examination revealed a nontender, elastic soft mass 30 x 15 centimeters in size (Figure 1).

**Figure 2:**
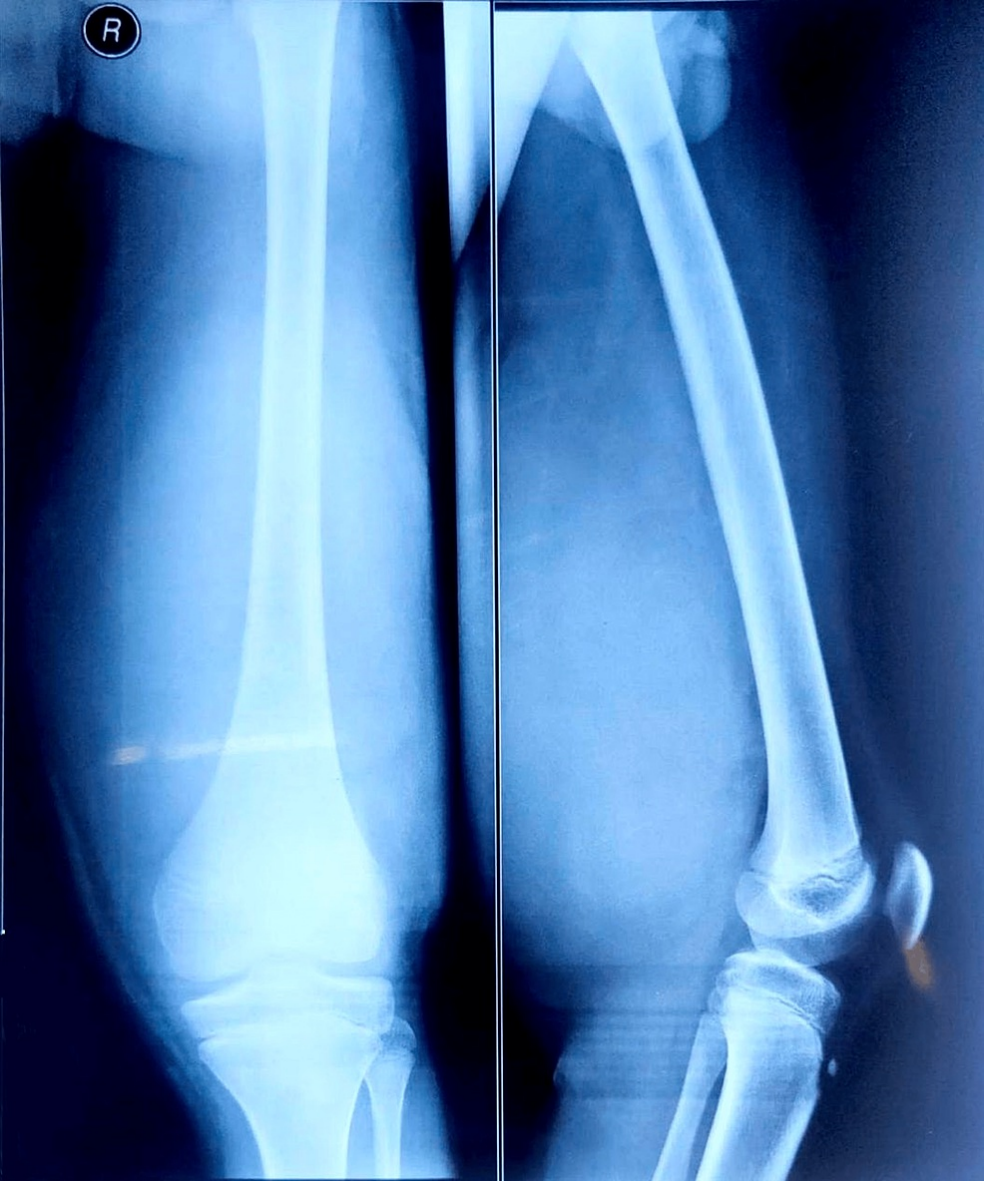
X-ray of right thigh showing extraskeletal mass

Laboratory tests revealed the value of alkaline phosphatase to be slightly high at 158 U/L. Leukocyte counts were normal (9300/L). Other biochemical tests were normal. Simple radiographs revealed an extra-skeletal mass in soft tissue at the posterior aspect of the right thigh on the anteroposterior view (Figure 2). Ultrasonography (USG) of the right thigh confirmed the presence of a sizeable soft-tissue mass measuring 26 x 9 cm. Soft tissue components showed multiple areas of necrosis and evidence of immense irregular vascularity. The underlying bone appeared normal. Magnetic resonance imaging (MRI) further exhibited a mass of size 26 x 9.5 x 14.7 centimetres extending into the adjacent proximal leg soft tissue with internal hemorrhagic and cystic components, which showed iso to hypointense signal on T1-weighted images. On T1-weighted images, the lesion revealed significant areas of hyperintense signal, indicating blooming on gradient recalled echo (GRE) images, and represented internal hemorrhagic components. The lesion had cystic components of fluid, which implied intensely hyperintense signals seen on the T2-weighted short Tau inversion recovery (STIR) images. These findings were neoplastic lesions that were most likely soft tissue sarcomas.

**Figure 3:**
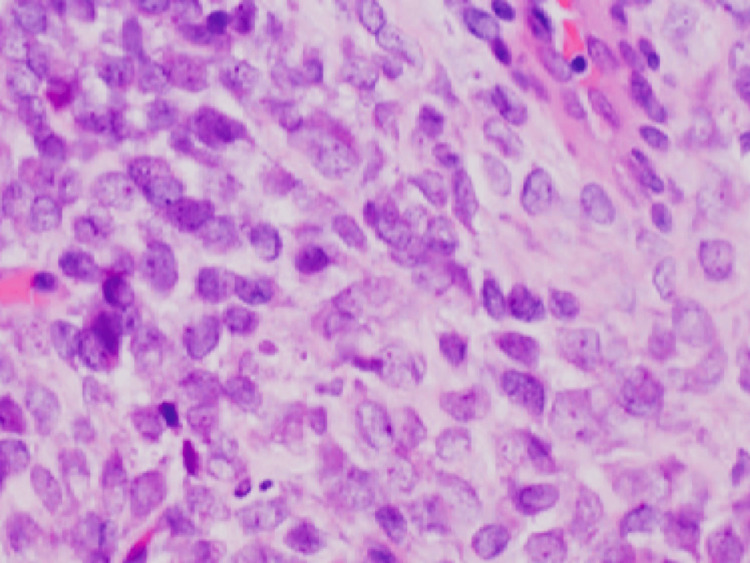
Histopathological image of the tumour showing densely packed tumour cells

Multiple soft tissue samples of the right thigh mass were taken with an 18-gauge needle to determine the histopathological diagnosis. Histopathological analysis revealed that the tumour included densely packed tumour cells arranged in diffuse solid sheets. Tumour cells were large, with round or oval nuclei, enlarged nuclei, stippled chromatin, and inconspicuous nucleoli with a scant amount of cytoplasm (Figure 3). Numerous mitoses with vascular proliferation were seen.

Immunohistochemically, tumour cells were reactive for CD99, vimentin, and cyclin D1 and negative for S100, desmin, and synaptophysin (Table 1). Overall, the histological features suggested malignant round cell sarcoma-Ewing's sarcoma. Using formalin-fixed, paraffin-embedded tissues, additional reverse transcription-polymerase chain reaction (RT-PCR)-based molecular analysis was done. RT-PCR found a fusion transcript of BCOR and CCNB3.

**Table 1:** Immunohistochemistry analysis CK: cytokeratins; EMA: epithelial membrane antigen; SMA: smooth muscle actin; SATB2: special AT-rich sequence-binding protein 2; WT-1: Wilms tumor-1; Tdt: terminal deoxynucleotidyl transferase; SS-18: synovial sarcoma-18; SOX10: SRY-related HMg-Box gene 10

IHC Markers	Result
CK	Negative
EMA	Negative
Desmin	Negative
SMA	Negative
S-100	Negative
SATB2	Few weak positive
WT-1	Negative
Tdt	Negative
Cyclin D1	Diffuse positive
SS-18	Negative
SOX10	Negative
ME-3	Retained
Ki67	Non contributory (repeated twice)
CD99	Positive

Following that, the team decided to start treatment with neoadjuvant chemotherapy, which included vincristine, doxorubicin, and cyclophosphamide on day one, followed by ifosfamide and etoposide on days one to five in accordance with the patient's weight, every 21 days. The patient received four cycles of chemotherapy, following which another MRI was performed. The patient experienced no other negative effects beyond hair loss. MRI results that evaluated the effectiveness of neoadjuvant treatment revealed that the tumour's size had decreased and now measured 23.3 x 6.5 x 10.7 centimetres.

**Figure 4:**
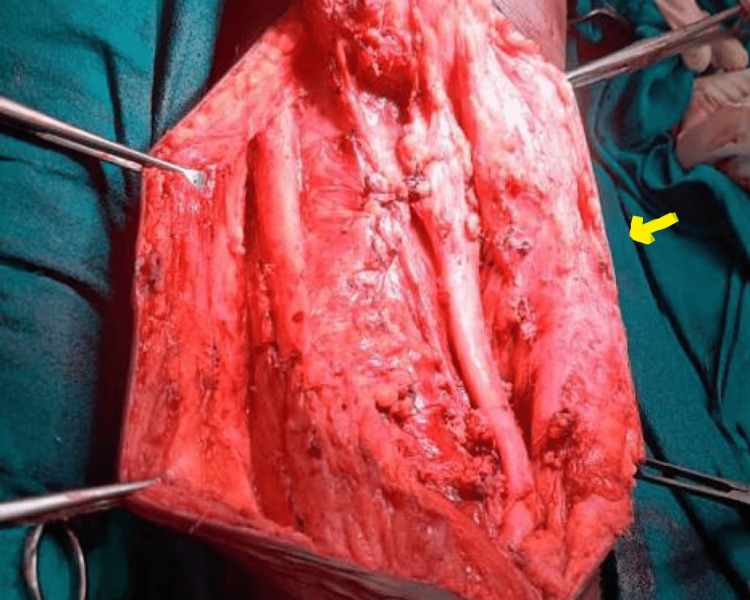
Resection of semitendinosus muscle showing greater sciatic nerve in the right (lateral side) The right side of the image is the lateral side (yellow arrow), the left side of the image is the medial side, the top being the cranial and the bottom being the caudal side. Anatomical structures that can be appreciated (right to left) are: greater sciatic nerve, cut part of biceps femoris, great saphenous vein

Following this, a limb-saving surgery was performed for a wide local excision. An incision reaching the subcutaneous tissue was made, and a skin flap was raised from both sides. After that, the semitendinosus muscle involved in malignancy was resected, the distal end was cut, and space was created, while the long head of the biceps femoris was resected near its origin (Figure 4).

**Figure 5:**
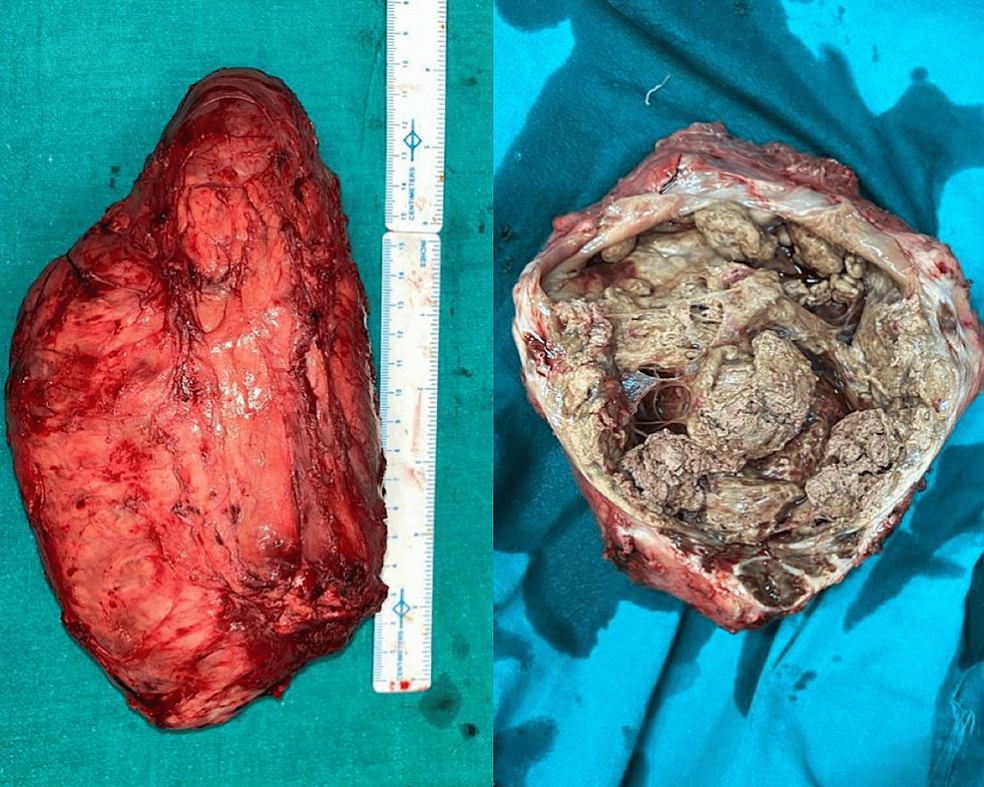
Resected mass

The great saphenous vein and semimembranous muscle were preserved. Then, sciatic nerve fibres adherent to the tumour were separated, while the nerve was preserved with great caution. The surgery was completed with the tumour being separated from all corresponding margins and excised. After resection, a specimen of the mass was taken and sent for histopathological analysis, which revealed only areas of necrosis and fibrosis with no residual tumour (Figure 5). The tumour was classified as T4-N0-M0 since the tumour mass was greater than 15 cm and no lymph nodes or metastases were seen.

A partial foot drop was discovered upon examination on the first postoperative day, but the patient was still able to walk, and the sutures were removed eight days later. Following the start of radiotherapy, the patient underwent two months of physiotherapy before walking normally and showing no signs of foot drop.

## Discussion

In 2012, Pierron et al. discovered the COR-CCNB3 fusion sarcoma, and in 2020, it was assigned to the new subgroup of undifferentiated small round cell sarcomas [1,4]. Due to the newly formed entity of sarcoma, many recent studies on BCOR-CCNB3 have re-diagnosed tumours that were previously called undifferentiated sarcomas by using genomic and molecular approaches. Even now, this sarcoma is an extremely uncommon tumour seen by orthopaedic specialists. The imaging characteristics of the aggressive malignant bone tumours, Ewing sarcoma and BCOR-CCNB3 sarcoma, are similar. The very limited number of such case reports and the dearth of case reports with imaging have made the characterization of radiological findings in BCOR-CCNB3 sarcomas difficult. However, a recent case series with literature analysis and a review article by Brady et al. and Sirisena et al. have documented specific imaging characteristics [9,10].

Bone BCOR-CCNB3 sarcomas frequently have uneven nuclei and proliferation of short, spindle-shaped to round cells as a primary histopathological feature [11]. Tumour cells from the bone cancer BCOR-CCNB3 are more likely to have a spindle-shaped morphology than those from a conventional Ewing sarcoma. According to reports, certain BCOR-CCNB3 sarcoma cases have stromal cellularity alterations and myxoid modifications (5,11). The most common bone locations affected are the long bones of the limbs (n = 28; 38.9%), the pelvis (n = 27; 37.5%), the calcaneus (n = 7; 9.7%), and the spine (n = 6; 8.3%). The femur (n = 13) is the most common position among the long bones of the limbs, followed by the tibia (n = 10) and fibula (n = 4). The metaphyseal-diaphyseal section of the femur or tibia is frequently affected by BCOR-CCNB3 sarcoma of the bone. These more frequent BCOR-CCNB3 sarcoma bone locations are similar to those that have been reported for skeletal Ewing sarcoma [12].

Radiological characteristics for BCOR-CCNB3 sarcomas of the bone are difficult to outline because of the relatively small number of cases that have been reported and the frequent occurrence of case series that do not contain imaging findings. Recent review articles by Brady et al. and Sirisena et al. reported the commonest imaging features to be bone lysis, sclerosis, mixed lysis, and lysis [9]. Radiology findings in our case showed no obvious bony erosions or destruction. According to the MRI findings evaluated by Sirisena et al., BCOR-CCNB3 sarcomas of the bone often showed intermediate signal intensity on T1-weighted imaging and heterogeneous elevated signal intensity on T2-weighted imaging [10]. Similar findings were seen in our case with iso- to hypointense signals on T1-weighted imaging and heterogenous hyperintense signals on T2-weighted imaging. Additionally, the presence of a tumour demonstrating extraosseous soft-tissue extension is a fairly common finding for most BCOR-CCNB3 sarcomas of bone, which is present in this instance as well. This finding can also be seen in Ewing sarcomas, making them difficult to distinguish. T2-weighted MRI of Ewing sarcoma, on the other hand, almost always reveals a uniform intensity, compared to the heterogeneous intensity seen in BCOR-CCNB3 sarcomas. Another differential for the current case could be osteosarcoma, which is the most frequently occurring primary malignant bone tumour in the age group of the first two decades of life and has similar findings in radiology as Ewing sarcoma. As there is no bone erosion or destruction in our case, this possibility can be ruled out.

The gold standard for soft tissue mass diagnosis is an open incisional biopsy, with a diagnostic accuracy of 94-99%. However, due to it being expensive, requiring general anaesthesia, and posing a significant complication rate of up to 16%, including hematoma, tumour spread, and wound problems, it may impede adjuvant treatments. While core needle biopsy (CNB) and fine needle aspiration cytology (FNAC) are relatively fast, convenient, cost-effective, minimally morbid, only require local anaesthesia, and pose a theoretically lower risk of local contamination, the inability to further identify the subtype of tumours limits the use of CNB and FNAC. [13]

Histopathological features of BCOR-CCNB3 sarcoma can vary from mostly spindle cell morphology to ovoid and angulated cells interspersed with areas of spindle cell morphology with scant cytoplasm, a pattern uncommon for Ewing sarcoma or CIC rearrangement sarcomas, which are characterised by uniformly small, round blue cells. Immunohistochemistry findings are consistent in most cases, with strong positivity for CCNB3 and a patchy, weak positivity for CD99. BCOR-CCNB3 fusion-associated sarcomas can be correctly diagnosed by paying attention to their distinctive appearance and using the appropriate immunohistochemistry markers. However, to establish the presence of BCOR-CCNB3 fusion, molecular genetic investigations such as fluorescence in situ hybridization (FISH), RT-PCR, or RNA sequencing are necessary for a definitive diagnosis.

The clinical results and treatment modalities for patients diagnosed with BCOR-CCNB3 are uncertain at this time due to the low number of people with this condition who have been documented to date and the variety of treatment regimens that have been delivered. To this day, neoadjuvant chemotherapy, followed by surgery and postoperative adjuvant chemotherapy, representing the same strategy employed for Ewing sarcoma, has been recommended as a standard treatment for BCOR-CCNB3 sarcoma. Our patient likewise underwent treatment following the aforementioned criteria, and as of eight months after surgery, there was no evidence of a recurrence. Most soft tissue and bone BCOR-CCNB3 fusion sarcomas that have been treated with Ewing chemotherapy regimens either before or after surgery have a five-year overall survival rate of 72% [8], which is higher than the survival rate for Ewing sarcoma.

## Conclusions

Due to its histological resemblance with Ewing sarcoma, BCOR-CCNB3 sarcoma necessitates distinction from Ewing sarcoma and small cell osteosarcoma using diagnostic imaging. The presence of patchy, slightly positive immunohistochemistry staining for CD99 and robust, diffusely positive immunohistochemistry staining for CCNB3 can help in the diagnosis of BCOR-CCNB3 sarcoma. For a final, definite diagnosis, RT-PCR confirmation of the BCOR-CCNB3 fusion gene is required. This report adds to our understanding of the BCOR effects on fusion-positive undifferentiated sarcomas at both the genomic and epigenomic levels, and it suggests that as better and even more refined treatment algorithms come up, epigenetic modifying entities should be tested for efficacy against these tumours.
